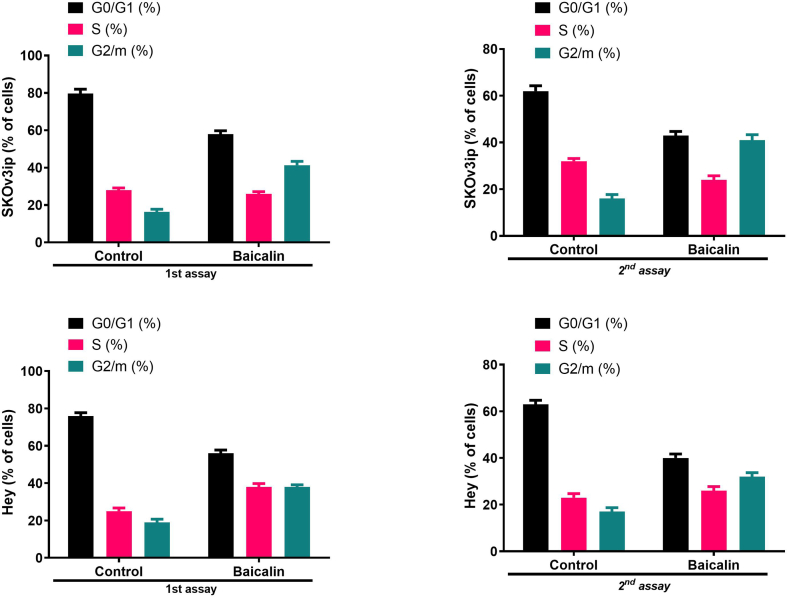# Chemoprotective effect of baicalin against cyclophosphamide induced ovarian toxicity in mice via inhibition of TGF-β

**DOI:** 10.1016/j.heliyon.2023.e22079

**Published:** 2023-11-16

**Authors:** Xuefeng Li, Juan Yue, Yogesh Kumar, Yannan Ma

**Affiliations:** a Department of Obstetrics and Gynecology, TangDu Hospital, Xi'an, 710000, China; b Department of Pharmacology, Akshita College of Pharmacy, Meerut, India; c Department of Gynecology, Xi'an International Medical Center Hospital, Xi'an, 710100, China

**Keywords:** Baicalin, Apoptosis, Ovarian toxicity, TGF-β, Inflammatory, Cell cycle G2/M arrest

## Abstract

Ovarian toxicity is most common gynecologically related malignancy, arising for most cases owing to the advanced stage of diagnosis. The aim of the current study was to explore the anticancer potential of baicalin against cyclophosphamide (CP) induced ovarian toxicity in mice and explore the possible mechanism. ovarian cancer cells (Hey, SKOv3ip and HO891PM) were treated with different doses of baicalin and examined via flow cytometry and cell proliferation assay. Subcutaneous administration of CP (200 mg/kg) was used to induce the ovary toxicity and mice were received the oral administration of baicalin. Oxidative, pro-inflammatory, inflammatory, apoptosis parameters, progesterone, estrogen hormones and histopathological were also estimated at end of the study. Baicalin increased the apoptosis and caused the cell cycle arrest at the G2/M stage in ovarian cancer cells. Baicalin significantly (P < 0.001) reduced the level of TGF-β in the HO8910PM, SKOv3ip and Hey cell lines. Baicalin significantly (P < 0.001) increased the body weight and reduced the tumor volume in mice. Baicalin significantly (P < 0.001) increased the level of estrogen and progesterone. Baicalin significantly (P < 0.001) reduced the level of malonaldehyde (MDA) and increased the level of superoxide dismutase (SOD) and catalase (CAT). Baicalin significantly (P < 0.001) decreased the level of pro-inflammatory cytokines such as tumor necrosis factor-α (TNF-α), interleukin-1β (IL-1β), interleukin-6 (IL-6) and inflammatory parameter such as nuclear kappa B factor (NF-κB), respectively. Baicalin significantly (P < 0.001) reduced the level of the caspase-3. Baicalin, act as the potential agent against the ovarian toxicity by alteration of TGF-β and inflammatory pathways.

## 1 Introduction

Ovarian cancer is considered the most dangerous gynecological malignancy, as it often occurs at an advanced stage by the time of diagnosis for most patients [1,2]. Ovarian cancer is considered the most prevalent type of cancer among women, particularly in the realm of gynecological neoplasms. Additionally, it ranks as the fifth leading cause of cancer-related deaths among women [3]. As a result, advancements in diagnosis and surgical options have led to improved outcomes. However, it is important to note that the 5-year survival rate for ovarian cancer in stages IIB to IV remains less than 40% [4,5]. Until now, the available treatments for ovarian cancer have been postoperative chemotherapy and surgical resection. Among the various chemotherapy agents used, platinum combined with paclitaxel is utilized as the first-line anticancer therapy for ovarian cancer. However, this therapy has limitations due to the development of drug resistance [6,7]. In recent years, researchers have been focusing their studies on developing novel therapeutics and understanding the underlying mechanisms of ovarian cancer [8,9]. On the contrary, the survival rate for ovarian cancer patients remains disappointing, primarily due to challenges such as chemotherapy resistance and recurrence despite available therapies [7,9]. Currently, researchers are directing their focus towards searching for novel chemotherapeutic agents that can effectively treat ovarian cancer while minimizing side effects.

It is well proofed that the cyclophosphamide (CP) is an alkalyting agent and commonly used to treat the various types of cancer. The toxic and therapeutic effects of CP depend on related active metabolites as phosphoramide and acrolein [10,11]. During the process, these metabolites bind with the DNA lead to disturbance of DNA synthesis and ultimately induce the cell death. CP metabolites generate the reactive oxygen species (ROS) via conjugation with glutathione resultant alteration the endogenous antioxidant defense system in the ovary. Previous research suggests that during CP treatment, there is an increase in the level of lipid peroxidation and a reduction in the level of SOD, CAT, GSH and other related factors [10,12,13]. CP induces the ovarian failure due to secretion of ovarian hormones and granulosa cell proliferation. The toxic effects of CP on the ovary include a decrease in the number of preantral, primordial, and antral follicles, which are associated with reduced levels of estradiol and progesterone hormones. During CP treatment in female mice, the number of primordial and growing follicles is reduced, ultimately leading to a decreased reproductive lifespan. Additional side effects of CP chemotherapy include infertility and amenorrhea [10,12,14].

The current approach for cancer prevention involves the use of plant-based drugs, isolated compounds from plants, dietary products, or synthetic compounds that serve to prevent or suppress the progression of malignancy. This approach has become increasingly popular due to the rising global incidence of cancer [[15], [16], [17]]. Some of the epidemiology studies show that the regular consumption of vegetable and food, which activate the anticarcinogens effect via decrease the harmful substances [18,19]. The regular intake of foods and vegetable increase the amount of beneficial nutrient viz., unsaturated fatty acids, flavonoids, selenium, terpenes, polyphenolic terpenoids etc. In this study, we used the baicalin (plant based drug) to treat the CP induced ovary dysfunction in mice.

## 2 Material & methods

### 2.1 Cell culture and reagents

The human ovarian cell lines Hey and SKOv3ip were obtained from the Anderson Cancer Center (Houston, USA). RPMI or DMEM medium (suspended in fetal bovine serum) was used for the culture the cell lines. The ovarian epithelial surface was lightly scraped to yield non-cancerous ovarian surface epithelial (OSE) cells, which were then cultured in medium 199:105 supplemented with 15 % foetal bovine serum and 10 ng/ml EGF (Sigma, St. Louis, MO), as previously described [20]. The Nanjing KeyGen Biotech Co., Ltd. (Nanjing, China) provided the human ovarian epithelial carcinoma cell lines (HO891PM), which were cultivated in RPMI-1640 media (Hyclone, Logan, UT, USA) supplemented with % FBS.

### 2.2 MTT assay

For the estimation of cell viability effect, we performed the methylthiazoltetrazolium (MTT) assay [21]. Each well contained 20 μL of 3-(4,5-dimethylthiazol-2-yl)-2,5-diphenyltetrazolium bromide (MTT, 5 mg/mL; Sigma-Aldrich, St. Louis, MO, USA), which combined with cells to produce Formazan precipitates. The liquid was removed, and each well received 150 μL of dimethyl sulfoxide (DMSO; Sigma-Aldrich) to dissolve the Formazan precipitates. At 490 nm, the absorbance (also known as optical density, or OD) of each well was measured to represent the vitality of the cells.

### 2.3 Estimation of cell proliferation

The ovarian cancer cell lines and OSE cells were seeded on the 96-well plates and incubated for 3 days with different baicalin concentrations (0–240 μM for OSE cells and 0–40 μM for cancer cell lines). Added 50 μl trichloroacetic acid (30%) and incubated again for 1 h at 4 °C. The cell lines were washed and dry, 100 μl solution of SRB (0.4 %) was added for ½ h. the acetic acid (01 %) was used for the washed the plate and air dried, after that Tris base (100 μl) was added, and the prepared plate was shaken for 5 min and the SRB value was estimated at the 490 nm wavelength [21,22].

### 2.4 Cell cycle analysis

Hey and SKOv3ip cellline were harvested in the 60 mm plates and incubated with baicalin (20–40 μM) and DMSO from the next day. The tested and control cells were trypsinized after the 24–48h treatment, collected in PBS and fixed on the ice, followed via raising with cold ethanol (70 %). After the treatment, the cells were stained with propidium iodide (50 μg/ml) at room temperature for 15 min. Flow cytometry was used for the estimation of cell cycle analysis of stained cells [23].

### 2.5 ELISA

Ovarian cancer cell lines were harvested in the plate (60 mm) and incubated with baicalin or DMSO. After 1, 2 and 3 days the culture supernatants were seed and the concentration of TGF-β1 was estimated. 96 well plates were used for harvested the Hey cells and incubated with the baicalin, or DMSO (Control) and Paclitaxel. SRB was used for the determination of the protective effect of baicalin on the proliferation of the ovarian cancer cell lines. ELISA reader was used for the estimation the concentration of TGF-β1 via using the available manufacturer's instruction.

### 2.6 Experimental rodent

Swiss albino mice (20 ± 5 g) sex-female was used in the current experimental study. The mice were received from the departmental animal house and kept in the standard laboratory condition (temperature 20 ± 5 °C, 60–70 % relative humidity, 12/12 h dark and light cycle). All the animal study was carried out according to the institutional animal protocol. The whole animal study was approved from the Xi'an International Medical Center Hospital (Approval:183791).

### 2.7 Toxicant and tested drug

The cyclophosphamide (toxicant drug) was used for the induction of ovarian toxicity. Subcutaneous administration of CP (200 mg/kg) was used for the induction of ovary toxicity [24]. The tested drug given to the mice orally in the form of suspension [25]. Oral suspension was prepared using the 1 % CMC suspension.

### 2.8 Experimental design

The mice were divided into different groups as follow and each group contains the 12 mice in each groups. Group I: normal control; Group II: cyclophosphamide (CP) control; Group III-IV: CP control treated with baicalin (1.25, 5 and 10 mg/kg) [25], respectively. The body weight and tumor volume of the experimental rodents were estimated at regular time intervals. At end of the current protocol, all the group mice anesthetized and collecting the blood samples via puncturing the retro orbital.

### 2.9 Progesterone and estrogen assay

Progesterone and estrogen level in the serum was estimated via using the ELISA kits following the manufacture instructions (Mouse Estrogen Bioassay, China). The quantity of progesterone and estrogen in the samples were estimated via using the standard curves and the amount was expressed in ng/l.

### 2.10 Antioxidant enzymes

The antioxidant enzymes such as superoxide dismutase (SOD), catalase (CAT) and malonaldehyde (MDA) were estimated using the ELISA kits following the manufacture instruction (Mouse Estrogen Bioassay, China).

### 2.11 Cytokines and inflammatory parameters

Cytokines parameters like tumor necrosis factor-α (TNF-α), interleukin-6 (IL-6), interleukin-1β (IL-1β), interlukin-10 (IL-10) and inflammatory parameters such as nuclear kappa B factor (NF-κB) were estimated using the (Nanjing Jiancheng Co., Nanjing, China) following the manufacture instruction.

### 2.12 Caspase

Caspase parameters were determined using the ELISA kit (Nanjing Jiancheng Co., Nanjing, China) following the manufacture instruction.

### 2.13 Statistical analysis

All the presented data were subject to statistical analysis and shown as the mean ± standard deviation. Two-tailed t-tests and data counting were evaluated using the chi-square criteria that measures parameter frequency.

## 3 Result

### 3.1 Cell viability effect

The effect of baicalin on cell viability was evaluated on different ovarian cancer cell lines, including HO8910PM (Fig. 1a), SKOv3ip (Fig. 1b) and Hey (Fig. 1c) at various time intervals. Baicalin demonstrated a dose-dependent reduction in cell viability against all types of ovarian cancer cell lines. The results indicate that baicalin inhibited cell viability across all time intervals. The IC50 values of baicalin against the various ovarian cancer cell lines are presented in Supplementary Table 1.

**Fig. 1:**
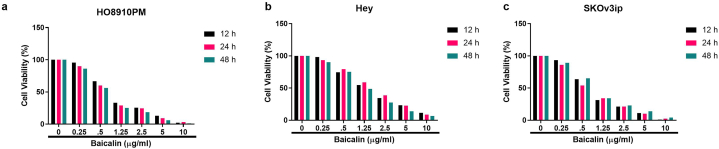
MTT assay was performed to estimation the viability of ovarian cancer cell incubated with baicalin for 12, 24 and 48 h. **a:** showed the viability effect on HO8910PM cells, **b:** showed the viability effect on SKOv3ip cells and **c:** showed the viability effect on Hey cells.

### 3.2 Effect of baicalin on CXCR4 expression

For the estimation of mechanism, we estimated the apoptosis effect of baicalin on the different ovarian cancer cell lines (data not showed). Baicalin treatment exhibited the reduced CXCR4 mRNA expression on the different cancer cell lines. Baicalin acid also decreased the protein level of CXCR4 and could be activated through SDF-1a in primary ovarian tumors and ovarian neoplasms (Supplementary Fig. 1).

### 3.3 Baicalin boosts cell adhesion

For the estimation of baicalin capacity to bind the extracellular matrix, we executed the cell adhesion assay using the ovarian cancer cell lines (HO8910PM, SKOv3ip, and Hey). Supplementary Fig. 2 showed that the adhesion of HO8910PM cells to matrigel was enhanced by 0.6 μg/μl of baicalin, while same concentration of baicalin on the adhesion of SKOv3ip and Hey cells was marginal.

### 3.4 Baicalin induce apoptosis and cell cycle arrest

Supplementary Fig. 3 showed the effect of baicalin on the cell cycle. The table showed that baicalin arrests the cell on the different cell cycle phase. Baicalin showed the increased increase in the G2.M phase on the SKOv3ip and Hey as compared to the untreated cells. Moreover, baicalin arrested ovarian cancer cells (SKOv3ip and Hey) in the G2/M phase.

### 3.5 Baicalin inhibit cancer cell migration

For the estimation of cancer cell migration, in vitro scratch assay performed. All the cells were pre-incubated in the FBS medium for 24 h before the cell proliferation. The wound healing effect was supervised at regular intervals till its completed closed. Supplementary Fig. 4 showed that the baicalin reduced the migration in the SKOv3ip, Ho8910PM and Hey.

### 3.6 Baicalin reduce TGF-β1 secretion

Baicalin was used for the estimation of inhibitory effect against the inflammatory mediators. Previously research suggests that the high concentration of TGF-β1 was observed in the cancer patient. Fig. 2a showed that the baicalin significantly inhibited the TGF-β1 concentration in the different ovarian cancer cells. Fig. 2b showed that the baicalin significantly reduced the cell growth and TGF-β1 secretion as compared to control.

**Fig. 2:**
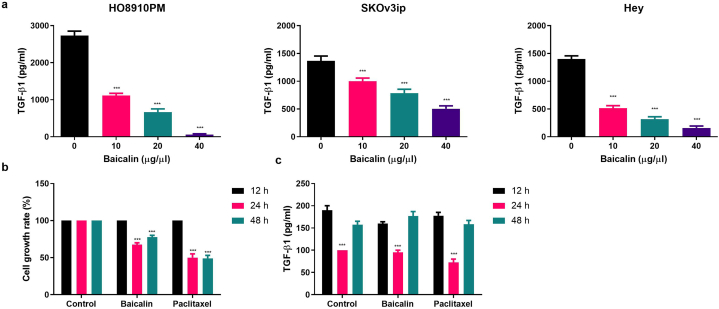
Baicalin reduces the secretion of TGF-β1 in ovarian cancer cells. **a:** dose dependent treatment of baicalin inhibited the secretion of TGF-β1 in all ovarian cancer cell lines, **b:** baicalin and paclitaxel reduced the cell growth. Experiments were repeated 3 times and *P < 0.05, **P < 0.01, ***P < 0.001 compared with untreated control cells).

### 3.7 Baicalin on ovarian cancer cell (*in vivo* model)

Fig. 3a showed the body weight of the different group mice, control and baicalin treated group mice. The tumor size of the mice was significantly reduced in the baicalin group mice, especially in the 10 mg/kg treated group mice (Fig. 3a).

**Fig. 3:**
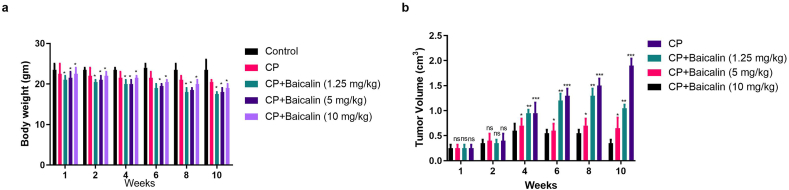
showed the effect of baicalin on cyclophosphamide induced ovarian cancer. **a:** showed the body weight effect at different time intervals and **b:** exhibited the effect of baicalin on tumor volume. All the data compared with control and *P < 0.05, **P < 0.01and ***P < 0.001as considered as the significant.

### 3.8 Effect on antioxidant parameters

Fig. 4 exhibited the effect of baicalin on the CP induced ovarian toxicity. CP induced ovarian toxicity group mice exhibited the increased level of MDA (Fig. 4a) and decreased level of CAT (Fig. 4b) and SOD (Fig. 4c) in the ovarian tissue and serum (Supplementary Fig. 5). Baicalin treated group mice showed the decreased level of MDA (Supplementary Fig. 5a) and increased level of SOD (Supplementary Fig. 5b) and CAT (Supplementary Fig. 5c) in the ovarian tissue and serum as compared to CP control group mice.

**Fig. 4:**
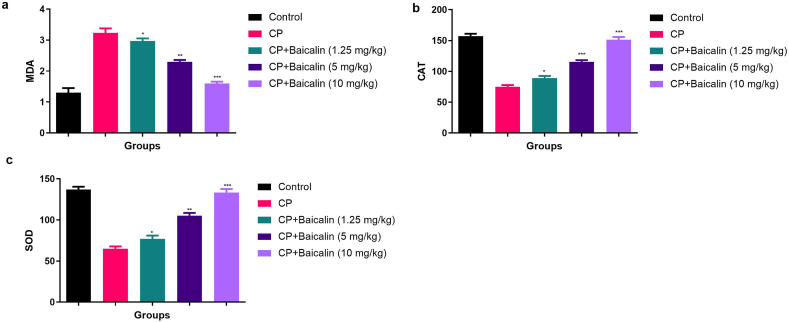
showed the effect of baicalin on the antioxidant parameter of cyclophosphamide induced ovarian cancer in the serum. **a:** showed the MDA level, **b:** SOD and **c:** CAT. All the data compared with control and *P < 0.05, **P < 0.01and ***P < 0.001as considered as the significant. Where MDA = Malonaldehyde, CAT= Catalase and SOD=Superoxide dismutase.

### 3.9 Effect on pro-inflammatory cytokines and inflammatory parameters

CP induced ovarian toxicity exhibited the increased level of pro-inflammatory cytokines such as TNF-α (Fig. 5a), IL-6 (Fig. 5b), IL-1β (Fig. 5c) and NF-κB (Fig. 5d) in the ovarian tissue and serum (Supplementary Fig. 6). Dose dependent treatment of baicalin significantly (P < 0.001) reduced the level of pro-inflammatory cytokines in the ovarian tissue and serum in the CP induced ovarian toxicity group mice.

**Fig. 5:**
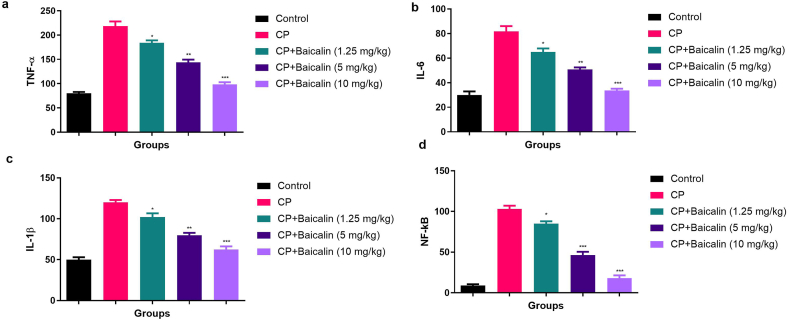
showed the effect of baicalin on the pro-inflammatory and inflammatory parameter of cyclophosphamide induced ovarian cancer in the serum. **a:** TNF-α, **b:** IL-6, **c:** IL-1β and **d:** NF-kB. All the data compared with control and *P < 0.05, **P < 0.01and ***P < 0.001as considered as the significant. Where TNF-α = Tumor necrosis factor-α, IL-6 = Interleukin-6, IL-1β = Interleukin-1β, NF-κB= Nuclear Kappa B factor.

### 3.10 Effect on caspase

Supplementary Fig. 7 showed the effect of baicalin on the caspase marker. CP induced group mice exhibited the increased level of caspase and dose dependent treatment of baicalin significantly (P < 0.001) down-regulated the caspase-3 activity.

### 3.11 Effect on estrogen and progesterone

During the ovarian toxicity, the level of estrogen (Fig. 6a) and progesterone (Fig. 6b) considerably reduced and similar result was observed in the CP control group mice. Baicalin significantly increased the level of estrogen and progesterone at dose dependent manner.

**Fig. 6:**
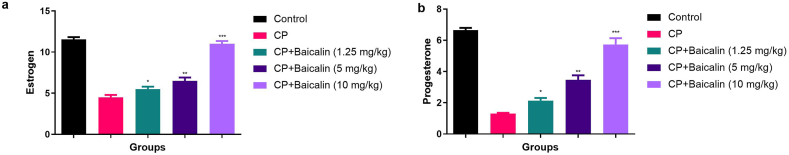
showed the effect of baicalin on progesterone and estrogen hormones of cyclophosphamide induced ovarian cancer. **a:** progesterone and **b:** estrogen. All the data compared with control and *P < 0.05, **P < 0.01and ***P < 0.001as considered as the significant.

### 3.12 Histopathology

**Fig. 7:**
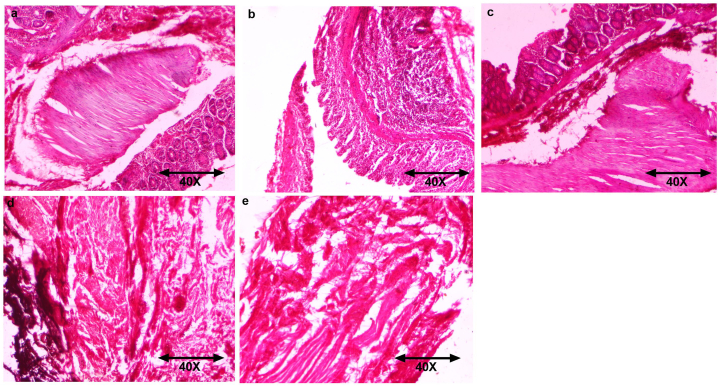
showed the histopathology of different group of mice.

Fig. 7 showed the histopathological figures of different group of rats. Fig. 7a exhibited the normal histopathology of ovary tissue. CP induced rats ovary tissue showed the hemorrhage, vascular congestion around the follicular atresia, corpus luteum and mononuclear cell infiltration (Fig. 7b). Rats treated with baicalin (figure c–e) restored the histopathological alteration.

## 4 Discussion

For chemotherapy, cyclophosphamide is the primary choice for the treatment of ovarian cancer. However, it is associated with serious side effects, and the development of resistance to cyclophosphamide poses a significant obstacle [26,27]. Another part, the researcher used the combination therapy for ovarian cancer [27,28]. The researcher chooses the combination the drug with the cyclophosphamide. But several cases the side effect does not decrease [26,29]. Currently, researchers are focusing on alternative systems of medicine for the treatment of ovarian cancer. Chemotherapy drugs like cyclophosphamide (CP) can cause severe damage to ovarian follicles, making them highly toxic to the ovaries. In a recent experimental study, we utilized CP at a dosage of 150 mg/kg to induce ovarian cancer. We then explored the potential benefits of baicalin, a plant-based drug, in improving the condition by increasing the levels of progesterone and estrogen. The study aimed to investigate the mechanism of action of baicalin on ovarian cancer cells and further analyze its effects.

*In vitro* ovarian cancer activity showed that the baicalin induced the cell cycle to seize at the G2/M stage and also increased the apoptosis [30]. We also observed the down regulating of Phospho weel, Cyclin B1, Phosphocdc2 and Myt1. It also showed the inhibition of TGF-β1 emission into the supernatant culture ovarian cell lines and also blocked Snail stabilization via TGF-β1. Several researchers targeting the G2/M inactivation, consequently they increased the DNA damage and latterly inducing the mitotic catastrophe and cell death in the cancer cell lines [31,32]. Cyclin B/cdc2 also play an important role in the maintaining the G2/M phase via speedily phosphorylating the marked protein to cause the development into the M phase. For controlling the G2/M cell cycle expansion via Cyclin B1/B2 complex play a significant role. In the G2 phase, phosphorylated cdc2 at Tyr15 and Thr14 via Wee1 and Myt1 protein kinase, thereby changing the G2 phase into an inactive precursor [33,34]. In the current experimental study, we found that baicalin reduces the protein content in Cyclin B1 and *p*-cdc2 in all cancer cell lines, which might exhibit the molecular level effect via inducing the cell arrest at G2/M phase. Its also confirmed reduced effect of p-Wee1 and Myt1 on all cell lines, showed the efficiency on baicalin in inducing the cell capture on G2/M phase. This effect is occurred due to inhibit the cdc2 and Cyclin B1 via Wee1 and Myt regulation.

CP causes the DNA damage in cancer cells and its enhance the survival of cancer patients, but also induce the side effect on the female reproductive system including temporary or permanent amenorrhea decreased infertility or fertility [10,11]. It is well documented that antral follicles and human primordial are sensitive to few drugs and down-regulation of follicles in ovary induce the permanent infertility. The main moiety of cyclophosphamide (Phosphoramide mustard) is the cytotoxic metabolite, its increase the ROS induce oxidative stress and increase the lipid peroxidation reaction and consequently leading to increase the antral follicle atresia and granulose cell apoptosis [10,35]. In the current experimental study, CP induced group mice exhibited the increased level of MDA (a lipid peroxidation marker) and reduced the level of SOD and CAT. These changes confirmed the induction of oxidative stress. Dose dependent treatment of baicalin significantly altered the level of endogenous antioxidant in the serum and ovarian tissue. Administration of antioxidant therapy is beneficial in protecting the fertility in women undergoing chemotherapy [11,35]. Other studies suggest that the inflammation play an important role in the development of ovarian toxicity. It is well documented that the inflammation is a physiological process in menstruation, implantation and ovulation, but uncontrolled inflammatory reaction has unfavorable effects on the hormones production and ovulation [10,14,36]. During the CP induce ovary toxicity; enhance the steroidogenesis and ovulation, which take part in the expansion of ROS. Its well proofed that ROS increase the inflammatory reaction and expansion the ovary disease. CP induced group mice exhibited that increase pro-inflammatory cytokines level in the serum and the ovary tissue. Baicalin treatment significantly reduced the level of pro-inflammatory cytokines level in the serum and ovary tissue. A similar effect was observed in the inflammatory mediators. On the basis of result, we can conclude that the baicalin reduced the ovary toxicity via antioxidant and anti-inflammatory effect.

Several researchers suggest that the TGF-β1 play an imperative role in the epithelial-mesenchymal transimition (EMT) and metastasis of ovarian cancer cells [37]. The researcher suggests that the ovarian cancer cells arising from the normal ovarian surface epithelium (OSE) [37,38]. Previous research suggests that the TGF-β1 is able to inhibit the OSE proliferation and also able to induce the apoptosis, which can able to escape the over-production of cancer cell during the normal ovulatory cycles [37,39,40]. During the early stage of tumor development, TGF-β1 act as the tumor suppressor via inhibiting the cell proliferation is also act as the metastasis promoter in various cancer cell model [38,39,41]. During the end stage of cancer, the cells can protect themselves and increasing the expansion of TGF-β1 from suppressor to promoter. Still, the mechanism of action of TGF-β1 is unknown, how to expand ovarian cancer, especially in the regulation of EMT [[37], [38], [39]]. Biochemical studies suggest that the concentration of TGF-β1 was higher found in the ovarian cancer suffering patient. Various research suggests that TGF-β1 is the crucial inflammatory factor during ovarian cancer [37,39]. Its also stabilize the protein content of snail, an inducer of EMT, and additionally increase the expression of a snail when it combined with the outer inflammatory mediators. In the current experimental study, baicalin exhibited the effect on the TGF-β1 at dose-dependent manner.

## 5 Conclusion

**Fig. 8:**
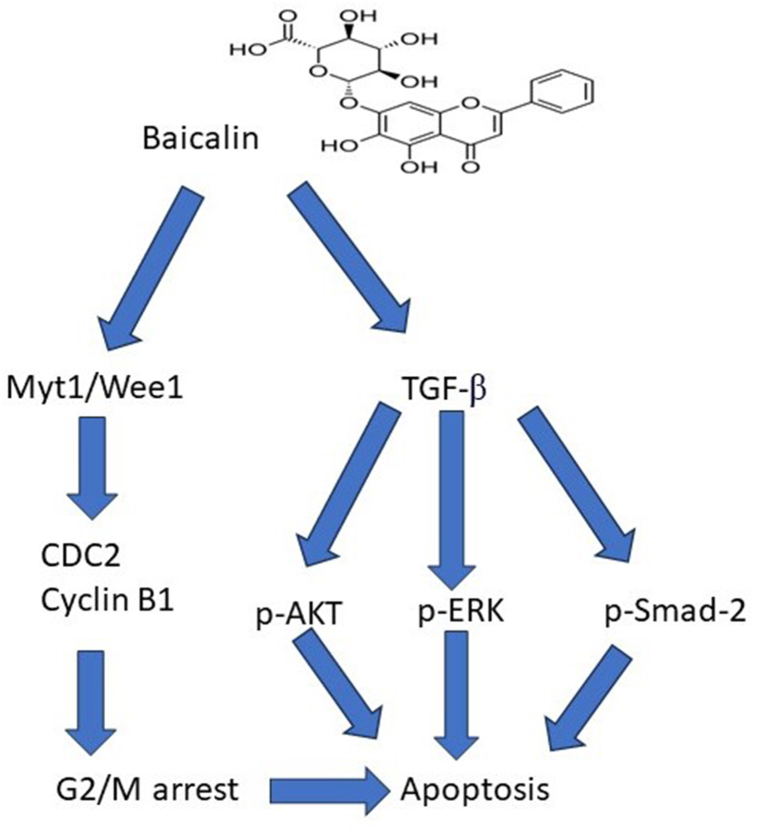
Underlying mechanism of action.

Our experimental study showed that the baicalein significantly reduced the progression of ovarian cancer cells (*in-vivo* and *in-vitro*), although it showed the low toxic effect against the normal cells. Baicalin reduced the secretion of TGF-β1 and also blocked the Snail stabilization that is caused by the TGF-β1. Baicalin also blocked the non-canonical ERK/AKT pathway and canonical Smad activation. Baicalin, by demonstrating the activity on the TGF-β/AKT/ERK/Smad signaling pathways (Fig. 8), serve as the main agent against ovarian cancer cells.

## Funding statement

This work was supported by Xi'an International Medical Center Hospital.

## Data availability statement

Data will be made available on request.

## Declaration of competing interest

The authors declare that they have no known competing financial interests or personal relationships that could have appeared to influence the work reported in this paper.

## Figures and Tables

**figs1:**
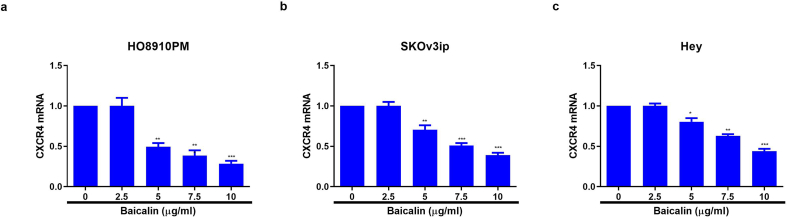


**figs2:**
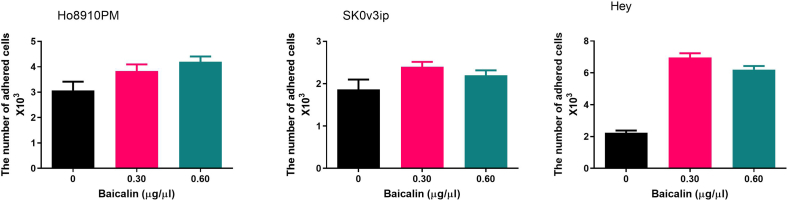


**figs3:**
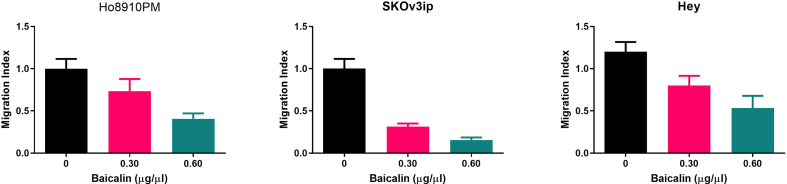


**figs4:**
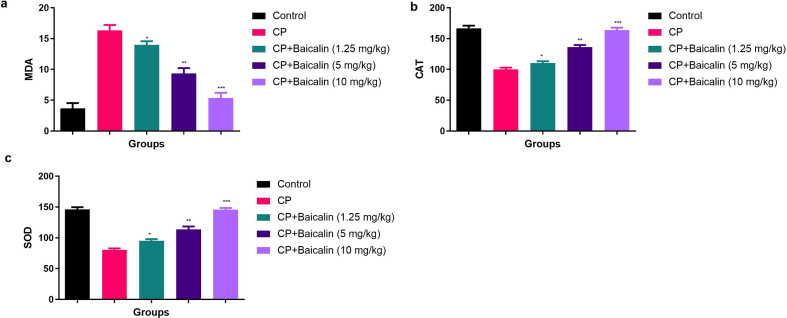


**figs5:**
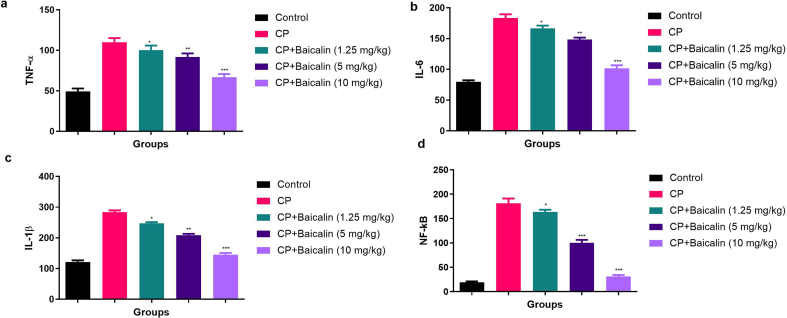


**figs6:**
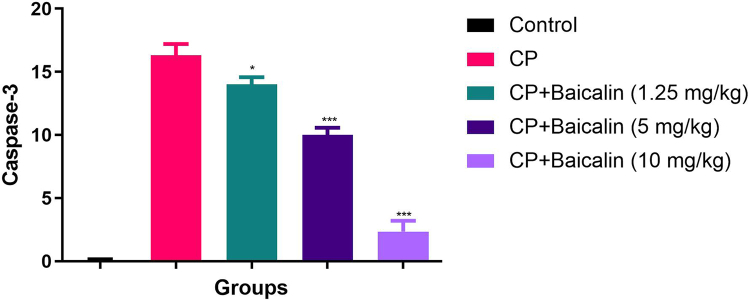


**figs7:**