# Supplementary material for: Chemoprotective effect of baicalin against cyclophosphamide induced ovarian toxicity in mice via inhibition of TGF-β
Source: Heliyon. 2023 Nov 16;9(12):e22079. doi: 10.1016/j.heliyon.2023.e22079 (PMC10716417; doi:10.1016/j.heliyon.2023.e22079)
Supplement: Multimedia component 1 [file mmc1.docx]

**Supplementary table 1:** Baicalin induces G2/M cell cycle arrest in SKOv3ip and Hey cells

| Cell lines |  | G0/G1 (%) | | S (%) | | G2/m (%) | |
| --- | --- | --- | --- | --- | --- | --- | --- |
|  |  | Control | AA | Control | AA | Control | AA |
| SKOv3ip | 1^st^ assay | 80 | 58 | 28 | 26 | 19 | 41 |
|  | 2^nd^ assay | 62 | 43 | 32 | 24 | 16 | 36 |
| Hey | 1^st^ assay | 73 | 56 | 25 | 38 | 19 | 38 |
|  | 2^nd^ assay | 63 | 40 | 23 | 26 | 17 | 32 |
